# Crimean-Congo Hemorrhagic Fever Case Series: a Chronology of Biochemical and Hematological Parameters

**DOI:** 10.7759/cureus.29619

**Published:** 2022-09-26

**Authors:** Said Amin, Fawad Rahim, Afsheen Mahmood, Huma Gul, Mohammad Noor, Asad Zia, Barkat Ali, Azhar Wahab, Urooj Khan, Furqan Ul Haq

**Affiliations:** 1 Internal Medicine, Khyber Girls Medical College, Peshawar, PAK; 2 Internal Medicine, Hayatabad Medical Complex Peshawar, Peshawar, PAK; 3 Virology, Khyber Medical University, Peshawar, PAK

**Keywords:** case series, blood group, alanine aminotransferase, thrombocytopenia, prognostic markers, high-sensitivity c-reactive protein, crimean-congo hemorrhagic fever virus

## Abstract

Introduction

Crimean-Congo hemorrhagic fever (CCHF) is a widespread tick-borne zoonotic disease. Sporadic outbreaks of CCHF occur in endemic regions, including Pakistan. The clinical spectrum of the illness varies from asymptomatic seroconversion to severe disease which may end in death. The treatment is supportive, including blood and blood products. There is multi-organ involvement in CCHF including acute hepatitis, thrombocytopenia, coagulopathy, acute kidney injury (AKI), and encephalopathy. Hematological and biochemical parameters may identify patients at substantial risk of worse outcomes. Early detection of the disease and forecasting the clinical course may be helpful. This case series aims to evaluate the trends of hematological and biochemical parameters among the survivors and non-survivors of CCHF.

Methods

All consecutive patients aged 16 years and above admitted to the isolation unit of Hayatabad Medical Complex, Peshawar, Pakistan between 1^st^ July and 30^th^ July 2022 with the diagnosis of CCHF were included in this case series. The diagnosis of CCHF was made by detecting viral ribonucleic acid by a polymerase chain reaction. For all patients, age, gender, address, occupation, clinical presentation, history of contact with animals, and travel history were recorded. All the vitals were taken regularly. The hematological (complete blood count) and biochemical parameters (serum creatinine, alanine aminotransferase (ALT), and C-reactive protein (CRP)) were documented daily. The blood group was determined for all the cases.

Results

Out of 17 cases, the majority (16 cases, 94.1%) were male and butchers (eight cases, 47.1%) by profession. All cases had significant contact with animals. Four patients (23.5%) died. Three out of the four non-survivors (75%) had ALT < 5 times the upper limit of normal with a static pattern of liver enzymes without much decline in ALT till death. One non-survivor (25%) had marked elevation of ALT at presentation, which had a declining trend till death. Seven out of 13 survivors (53.8%) had moderate to marked elevation in the level of ALT at presentation. The ALT showed a downward trend during the course of illness in all these patients. The remaining survivors (six out of 13, 46.2%) had a mild elevation of ALT and 50% of them showed improvement in the ALT level during hospitalization. All patients had thrombocytopenia at presentation. None of the non-survivors showed a persistent increase in the platelet count, and three cases remained severely thrombocytopenic at the time of death. However, the trend in platelet count among all the survivors was increasing. The CRP level in the majority (three out of four cases, 75%) of the non-survivors remained elevated till death, while all survivors showed a progressive decline in CRP level. A majority (11 out of 17 cases) had blood group B. Half of the non-survivors (two out of four cases) and the majority of the survivors (nine out of 13 cases) had blood group B. AKI was found in all non-survivors, while all the survivors had normal renal function throughout the course.

Conclusion

A persistently raised ALT and CRP level, a persistently low or decreasing platelet count, and AKI were associated with mortality. Blood group B was the commonest blood group among patients of CCHF, which is not reflective of the blood group distribution of the general population from which this case series has been reported.

## Introduction

Crimean-Congo hemorrhagic fever (CCHF) is a geographically widespread tick-borne zoonosis. The virus of CCHF was identified in 1967 in a patient in Uzbekistan and was found to be like a virus isolated in 1956 in Congo, hence the name CCHF virus [1]. Pakistan reported the first human case in Rawalpindi in 1976. Since 2000, at least 14 sporadic outbreaks have been reported in Pakistan [2].

The clinical spectrum of the illness varies from asymptomatic seroconversion and mild infection to severe disease and may lead to death. In severe cases, hemorrhagic manifestations develop 3 to 6 days after the onset of symptoms [3]. The reported fatality rates range from 4 to 20%, depending on the area and quality of medical care [4].

Early prediction of the clinical course of the disease may be lifesaving. Clinicians need to be aware of the clinical and laboratory features of CCHF patients that make it possible to predict the future course of illness, plan appropriate management, and shift the patient to a healthcare facility on time for specialized treatment [5]. The disease course is guided by clinical signs such as Glasgow coma scale (GCS) score, mucocutaneous bleeding, and hemodynamic instability. Thrombocytopenia, leukopenia, coagulopathy, and elevated liver enzymes are the primary hematological and biochemical abnormalities [6]. Increased mortality has been reported with increased viral load, thrombocytopenia, prolonged prothrombin time (PT) and activated partial thromboplastin time (APTT), leukocytosis, elevated alanine aminotransferase (ALT), elevated lactate dehydrogenase (LDH), low fibrinogen, and low GCS score [1].

In the Muslim world, it usually causes sporadic outbreaks during the Eid Ul-Adha festival due to contact with the animals sacrificed for religious rituals. Eid Ul-Adha festival was celebrated from the 10th to the 12th of July 2022. About eight million animals, including cows, sheep, goats, and camels, were slaughtered during Eid Ul-Adha in Pakistan only [7].

Advance purchase of animals to be sacrificed during Eid‐Ul‐Adha is a common practice in Pakistan. Most of these animals are placed in residential areas, preferably at homes. Moreover, people prefer the self‐slaughtering of animals due to the unavailability of professional butchers during the festivity and partly due to religious beliefs. These practices facilitate the animal‐to‐human transmission of the disease and the emergence of sporadic outbreaks [8].

Despite the rapidly growing incidence of the disease, there are currently no accurate data on the distribution, prevention, and control strategy for CCHF. Moreover, there is a knowledge gap in the pathogenesis and clinical course of the disease and the sequential changes in the markers of organ dysfunction [9]. The exact dynamics of the prognostic parameters in the CCHF disease course are however unpredictable and inconsistent [10]. We report a large case series of patients with CCHF to follow the chronological trends and interplay of hematological and biochemical parameters among the survivors and non-survivors.

## Materials and methods

All patients aged 16 years and above with a diagnosis of CCHF confirmed by serum polymerase chain reaction (PCR) admitted between 1st July and 30th July 2022 to the isolation unit of Hayatabad Medical Complex, Peshawar, Pakistan, were included in this case series. The reference laboratory of Khyber Medical University, Peshawar, Pakistan conducted all the PCRs. All cases were notified to the National Institute of Health, Pakistan. The institutional/ethical review board of Khyber Girls Medical College, Peshawar, Pakistan approved the case series. Informed consent was obtained from all the study participants.

The demographic parameters, clinical presentation, history of contact with animals, travel history, and professional details were recorded. All the vitals (blood pressure, pulse, temperature, and oxygen saturation) were recorded regularly. The hematological parameters (complete blood count) and biochemical parameters (serum creatinine, ALT, and C-reactive protein (CRP)) were documented daily. A blood group was ascertained for all the cases. Additionally, all the patients were screened for the malarial parasite, dengue fever, hepatitis A through E viruses, and Leptospira.

Degree of thrombocytopenia was defined as mild (101 x 10^3^ to 140 x 10^3^/mcL), moderate (51 x 10^3^ to 100 x 10^3^/mcL), severe (21 x 10^3^ to 50 x 10^3^/mcL), and very severe (<20 x 10^3^/mcL) [11]. Acute hepatitis was defined as ALT more than five times the upper limit of the normal range (ULN). Moreover, liver involvement was categorized as mild (ALT < 5 times ULN), moderate (ALT between 5 to 10 times ULN), and severe (ALT > 10 times ULN) [12]. The increase in CRP was categorized as mild (0.3 to 1 mg/dL), moderate (1 to 10 mg/dL), and marked (>10 mg/dL) [13]. Acute kidney injury (AKI) was defined as serum creatinine of more than 1.5 mg/dL at any point during the illness.

Clinical outcome was classified as survivors and non-survivors. We evaluated the trends of platelet count, ALT, CRP, creatinine, and blood group distribution among the survivors and non-survivors. All patients were offered standard supportive treatment, including blood and blood products, meropenem (1 g every 8 hours) (when sepsis was strongly suspected on clinical grounds), tranexamic acid (10 mg/kg every 8 hours), and ribavirin (30 mg/kg loading dose, then 15 mg/kg every 6 hours for 4 days, and then 7.5 mg/kg every 8 hours for 6 days).

## Results

**Figure 1:**
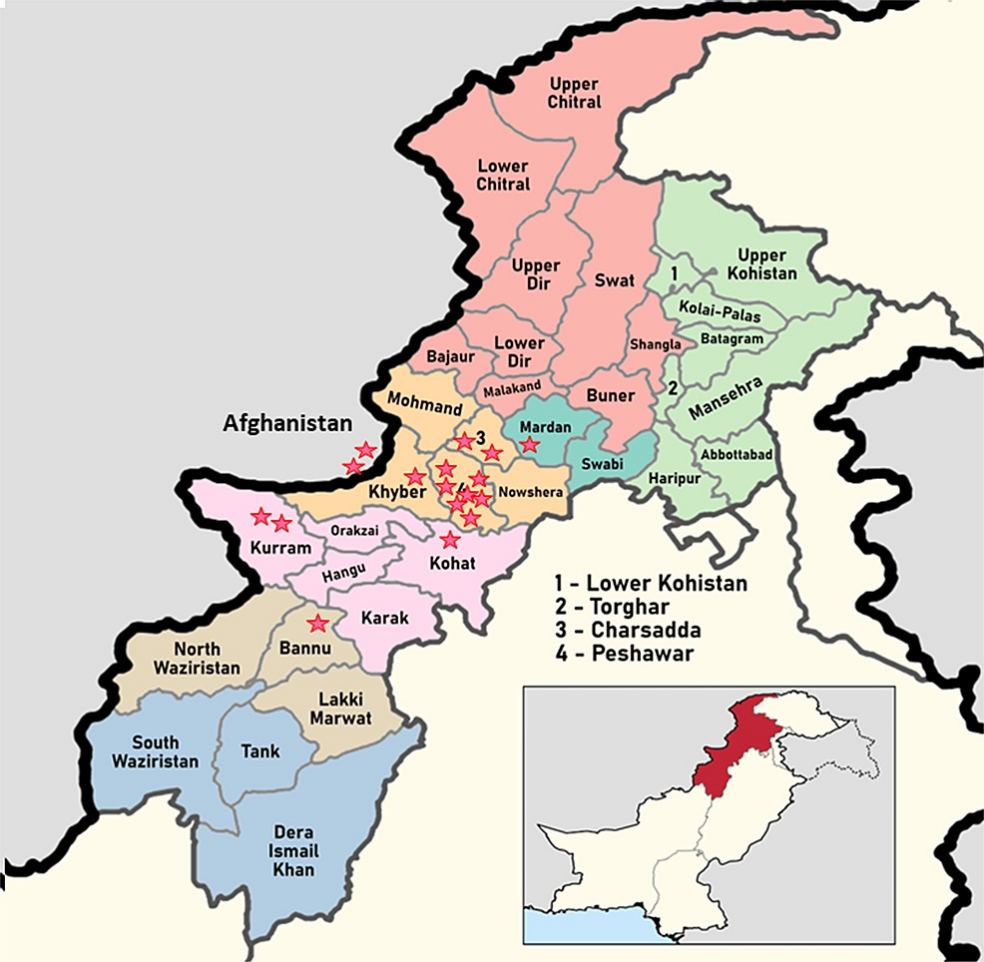
Map of Khyber Pakhtunkhwa province of Pakistan showing the distribution of cases of Crimean-Congo hemorrhagic fever.

A total of 17 PCR-positive CCHF patients were included in this case series. There were 16 (94.1%) male patients. Out of 17, 13 (76.4%) patients survived. Eight cases were butchers by profession, and three each were farmers and laborers. All had significant contact with animals in the form of catering and slaughtering of animals or visits to animal markets during the religious festivity of Eid-Ul-Adha. The geographical distribution of cases is shown in Figure 1.

**Figure 2:**
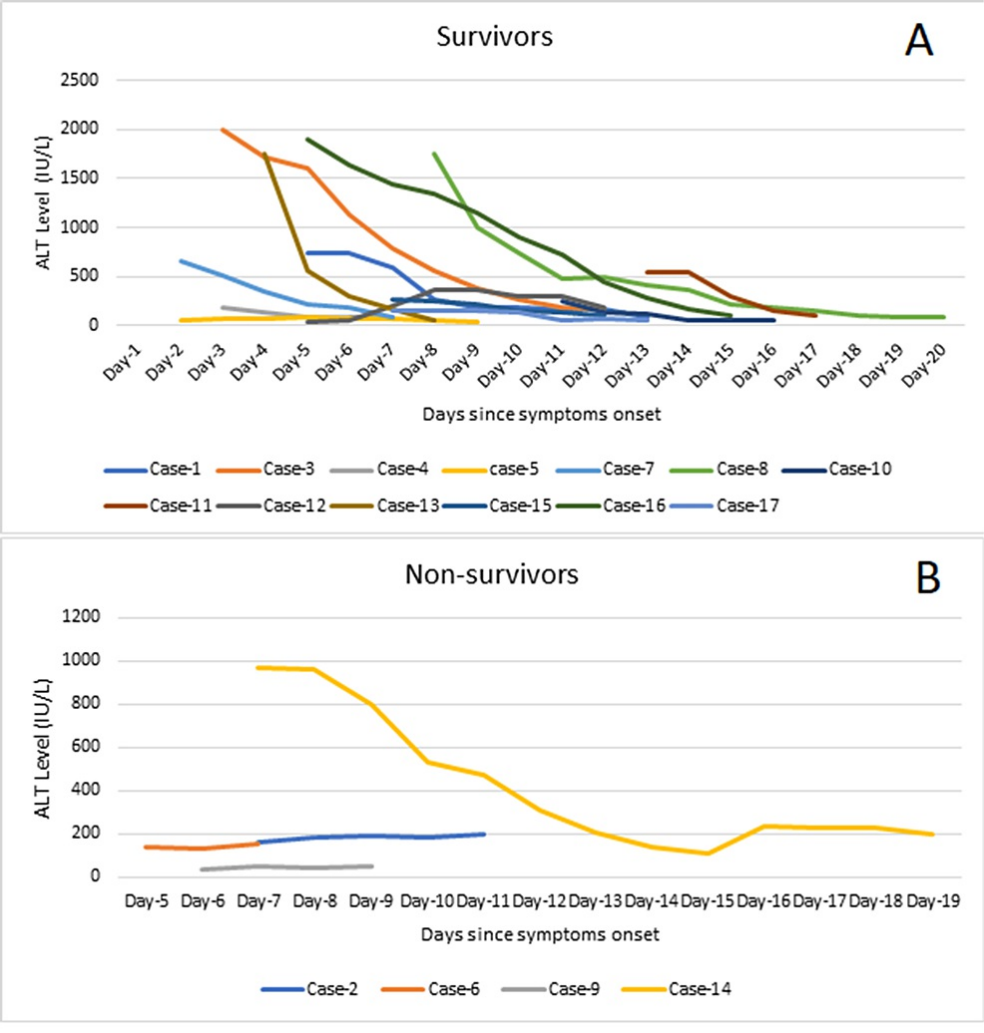
Trends of alanine aminotransferase (ALT) levels among the survivors (A) and non-survivors (B). ALT: alanine aminotransferase; IU/L: international unit per liter

In this case series, three out of the four non-survivors (75%) had up to mild liver involvement with a static pattern of ALT without much decline in the ALT till death. One non-survivor (25%) had marked elevation of ALT at presentation, which had a declining trend till death. Seven out of 13 survivors (53.8%) had moderate to marked liver involvement at presentation. The ALT showed a downward trend during the course of illness in all these patients. The remaining survivors (six out of 13, 46.2%) had mild liver involvement and 50% of them showed improvement in the ALT level during hospitalization (Figure 2).

**Figure 3:**
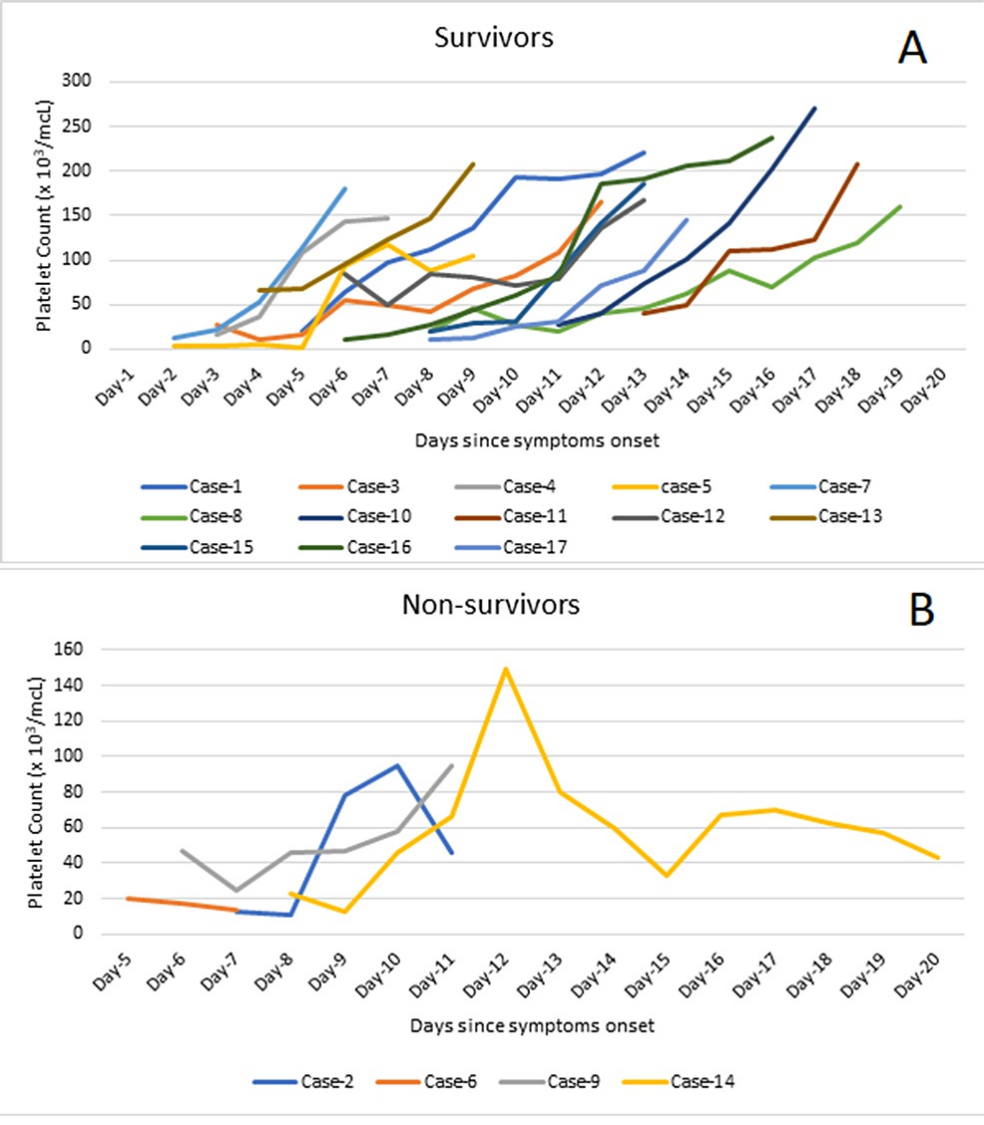
Trends of platelet count among the survivors (A) and non-survivors (B). mcL: microliter

Thrombocytopenia was noted in all cases on admission. Half of the non-survivors (two out of four) had severe thrombocytopenia while the rest had very severe thrombocytopenia at presentation. None of the non-survivors showed a consistent increase in the platelet count despite platelet transfusions, and three cases remained severely thrombocytopenic at the time of death. Six out of 13 (46.2%) survivors had very severe thrombocytopenia, five (38.4%) had severe thrombocytopenia, and two (15.4%) had moderate thrombocytopenia. All survivors had a progressive upward trend in the platelet count with transfusions, achieving a safe level (>100 x 10^3^/MCL) at the time of discharge (Figure 3).

**Figure 4:**
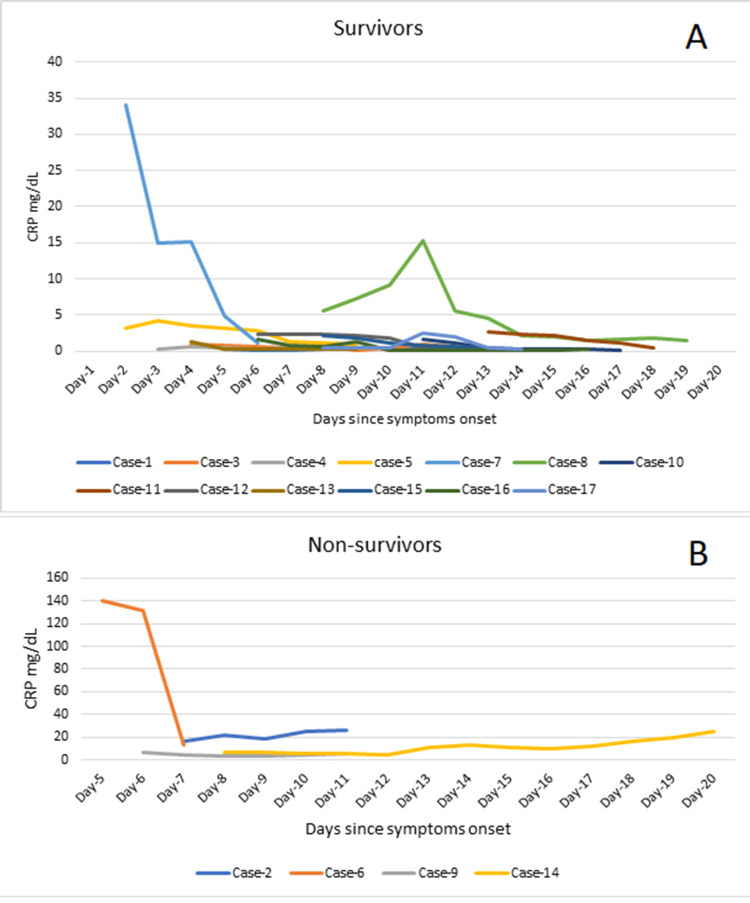
Trends of C-reactive protein (CRP) level among the survivors (A) and non-survivors (B). CRP: C-reactive protein; mg/dl: milligram per deciliter

The CRP level increased progressively in two (50 %) out of the four non-survivors till death while it decreased in one (25%) and stayed static in one (25%) non-survivor. Of the 13 survivors, nine (69.2%) presented with moderate, one (7.70%) with mild, and one (7.70%) with marked elevation of CRP while two (15.4%) survivors had normal CRP at admission. All survivors had a progressive decline in the CRP level till discharge (Figure 4).

A majority (11 out of 17 cases) had blood group B. Half of the non-survivors (two out of four cases) and the majority of the survivors (nine out of 13 cases) had blood group B. AKI was found in all four non-survivors, while all 13 survivors had normal renal functions throughout the course.

## Discussion

CCHF is endemic in the Eastern Mediterranean region, and in recent years, CCHF cases have been reported in Sudan (2010), Iraq (2010), Iran (2003,2012), Afghanistan (2008, 2012), and Pakistan (2017,2000,2012) [14].

This is one of the largest case series, having 17 PCR-positive CCHF patients. Four patients (23.5%) succumbed to this lethal disease. Out of 17, 16 (94%) patients were male. Eight cases were butchers by profession, and three each were farmers and laborers. All had significant contact with animals.

Deranged liver parameters are one of the most expected findings in hemorrhagic viral infections, usually manifested as raised bilirubin and transaminases. The severity of the liver injury depends on the magnitude of ALT elevation [12]. The trend of ALT in this case series highlights that a declining pattern of ALT has a good prognostic value compared to the actual level of ALT at presentation. Our results are parallel with the observations by Ayatollahi et al. from Iran and Muco et al. from Albania, who found a steep decline in the ALT among the survivors [15,16]. However, the non-survivors were not reported for comparison in the aforementioned case series. In contrast, Ahmeti et al. found no associa­tion of ALT with the outcome [17].

Thrombocytopenia and platelet function are poor prognostic indicators of CCHF [18]. The trend of platelet count in this series indicates that a consistent rising pattern of platelet count is associated with a favorable outcome. This finding is consistent with a study by Ekiz et al. from Turkey who observed a positive correlation between platelet count and mortality [19]. Swanepoel et al., Doğan et al., and Leblebicioglu et al. have also reported comparable findings [4,20,21].

An elevated CRP is an independent biochemical marker of severity in CCHF [1]. The course of CRP in this case series signifies that a decline in CRP level is linked with better survival while a progressive rise may predict a poor outcome. Yilmaz et al. and Buyuktuna et al. have also reported high CRP at admission as a poor prognostic indicator [10,22].

Studies have demonstrated that blood groups are associated with vulnerability to a wide variety of infections such as *Helicobacter pylori* (Blood group O), *Salmonella typhi* (Blood group B), Pseudomonas (Blood group A), and severe acute respiratory syndrome coronavirus-2 [23]. Blood groups might also be associated with coagulation proteins, and this association is clinically significant in viral hemorrhagic fevers [24].

In this case series, blood group B was predominant at 64.7% (11 out of 17 cases) overall and in 50% of the non-survivors. Güven et al. from Turkey reported blood group O as the predominant blood group in CCHF patients. It is pertinent to mention that blood group O is the second most widely distributed blood group after blood group A in the general population of Turkey [25]. In comparison, blood group B accounts for 31.65% of the blood groups of the general population of Khyber Pakhtunkhwa province, from where this case series has been reported [26]. The higher frequency of blood group B among patients with CCHF than in the general population could be indicative of increased susceptibility of blood group B to the CCHF virus. This warrants further research to determine the differential susceptibility of blood groups to the CCHF virus.

AKI was found in all four non-survivors, while all 13 survivors had normal renal functions throughout the course, signifying the importance of AKI as a predictor of mortality.

Given the single-center nature of the study and a sample derived from a predominantly Pashtun population, the results may not reflect the trends in the population at large. Prospective studies on a larger and geographically diverse sample are limited by the very nature of the disease which occurs only as sporadic outbreaks for a limited time in the Muslim world, usually around Eid Ul-Adha. Moreover, we could not determine the viral load of the CCHF virus due to resource constraints. Therefore, the interplay between the degree of viremia and hematological and biochemical parameters could not be studied.

## Conclusions

In this case series, 23.5% of patients did not survive. A persistently raised ALT and CRP level and a persistently low or decreasing platelet count were associated with non-survival in patients with CCHF. In addition, AKI was a common abnormality observed among the non-survivors. Blood group B was the commonest blood group among the patients of CCHF. The trends of the above biochemical and hematological parameters will assist the physicians to identify and effectively triaging patients with CCHF to the most appropriate level of care on time.
